# Translation and cross-cultural adaptation of the Nordic Occupational Skin Symptoms Questionnaire to Brazilian Portuguese

**DOI:** 10.47626/1679-4435-2022-959

**Published:** 2024-08-05

**Authors:** Ivana Gonçalves Labadessa, Andrea Antunes Cetlin, Rodolfo Schoma de Paula Machado, Flávia de Lima Osório, Rosângela Villela Garcia, Marcelo Bezerra de Menezes, Elcio Oliveira Vianna

**Affiliations:** 1 Faculdade de Medicina de Ribeirão Preto, Universidade de São Paulo, Ribeirão Preto, SP, Brazil

**Keywords:** entrevista, dermatite atópica, urticária, tradução, doenças profissionais, inquéritos e questionários, interview, dermatitis, atopic, urticaria, translating, occupational diseases, surveys and questionnaires

## Abstract

**Introduction:**

Occupational skin diseases are a frequently self-reported condition in industrialized countries. However, there are few developed and standardized self-report instruments to screen the population at risk for occupational dermatological diseases.

**Objectives:**

Translation and cross-cultural adaptation of the long and short versions of The Nordic Occupational Skin Symptoms Questionnaire into Brazilian Portuguese.

**Methods:**

The process of translation and cross-cultural adaptation of the questionnaire was developed following the good practice recommendations of the International Society for Pharmacoeconomics and Outcomes Research.

**Results:**

After translation into Brazilian Portuguese, the first reconciled version of the questionnaire was evaluated in a first round of interviews with 28 individuals, including patients with dermatological disease and healthy people. In the first meeting of the study review group, changes were made to 43 questions (75.4%) (e.g., inclusion of definition of terms, reformulation of instructions, and changes to alternative words or synonyms). In the second meeting of the study review group, there were modifications in three questions, creation of the second consensus version in Brazilian Portuguese, and then the back-translation of this version. After the second round of cognitive interviews, which took place with 10 patients, we had the third review group meeting (no modification was made) and definition of the final version of the questionnaire.

**Conclusions:**

The short and long versions of the Nordic Occupational Skin Symptoms Questionnaire questionnaire are available in Brazilian Portuguese.

## Abstract (pt)

**Introdução:**

As doenças ocupacionais de pele são uma condição frequentemente autorrelatada em países industrializados. No entanto, existem poucos instrumentos de autorrelato desenvolvidos e padronizados para triagem da população de risco para doenças ocupacionais de pele.

**Objetivos:**

Tradução e Adaptação Transcultural da versão longa e curta do Nordic Occupational Skin Symptoms Questionnaire para o português brasileiro.

**Métodos:**

O processo de tradução e adaptação transcultural do questionário foi desenvolvido seguindo as boas práticas recomendadas pela International Society for Pharmacoeconomics and Outcomes Research.

**Resultados:**

Após a tradução para o português brasileiro, a primeira versão conciliada do questionário foi avaliada em uma primeira rodada de entrevistas com 28 indivíduos entre pacientes com doença dermatológica e pessoas saudáveis. Na primeira reunião do grupo de revisão do estudo, foram feitas alterações em 43 questões (75,4%) (por exemplo, inclusão de definição de termos, reformulação de instruções e alteração para palavras alternativas ou sinônimos). Na segunda reunião do grupo de revisão do estudo, houve modificações em três questões, criação da segunda versão de consenso em português brasileiro e, posteriormente, a retrotradução desta versão. Após a segunda rodada de entrevistas cognitivas, que ocorreram com 10 pacientes, tivemos a terceira reunião do grupo de revisão (não houve modificação) e definição da versão final do questionário.

**Conclusões:**

As versões curta e longa do questionário Nordic Occupational Skin Symptoms Questionnaire estão disponíveis em português brasileiro.

## INTRODUCTION

Occupational dermatoses (OD) are a frequent condition in industrialized countries, and some studies have shown an increase in its prevalence in the last decades. However, there are few tools developed and standardized specifically for patients with OD that may contribute to the implementation of a screening approach.^1-3^

A study conducted with 630 patients (mean age 44.5 years, 64% men) from the allergy division of the dermatology clinics of a hospital in São Paulo, Brazil, found that 10.9% had occupational contact dermatitis. The frequency of ODs in the study was high among domestic workers (39%), construction workers (33.5%), metallurgists (6%), carpenters (4%), and hairdressers (4%); furthermore, 91.5% of patients with occupational contact dermatitis worked in a humid environment.^4^

Prevention action plans to avoid occupational diseases are important, and an early screening instrument is essential to implement effective prevention measures. Early screening also ensures diagnosis of diseases in an early stage, when treatment is likely to be most beneficial. It also helps to identify work-related and non-work-related exposures that increase the risk of illness (e.g., allergens, working with gloves, etc.).^5^

The Nordic Occupational Skin Questionnaire (NOSQ-2002) was developed in 2002 by a group of researchers from five northern European countries, known as the Nordic Occupational Skin Questionnaire Group, and its short and long versions are standardized and internationally recognized instruments for the assessment of occupational and non-occupational risk factors, and also for determination of the prevalence and the course of relevant occupational skin diseases.^6,7^

The original English version has already been translated into languages such as Danish, Finnish, Icelandic, Swedish, German, Spanish and Catalan,^1,5^ granting the possibility of epidemiological data comparison among studies and countries.^6^ However, the use of this questionnaire in Brazil is limited, since there is no Brazilian Portuguese version so far.

The aim of this article is to report the process of translation and cross-cultural adaptation of the long and short versions of the NOSQ-2002 into Brazilian Portuguese.

## METHODS

### ASPECTS OF THE QUESTIONNAIRE (NOSQ-2002)

As already mentioned, the Nordic Occupational Skin Questionnaire Group developed a short (S) and a long version (L) of the questionnaire (NOSQ-2002/S and NOSQ-2002/L), refined based on pre-existing experiences and questionnaires to be applied to the general population.^6^

The NOSQ-2002/S version consists of 13 questions divided into four categories (1- general: demographics and occupational history; 2- history of atopic symptoms; 3- self-reported eczema on hands or forearms; and 4- exacerbating factors) and can be used for screening and monitoring of hand and forearm eczema (e.g., in a population with a particular profile or in a particular workplace). The NOSQ-2002/L version consists of 57 questions grouped into 10 categories (1- general: demographic data and occupational history; 2- history of atopic symptoms; 3- self-reported eczema on hands or forearms; 4- exacerbating factors; 5- consequences and impact of skin diseases; 6- selfreported contact urticaria on hands or forearms; 7- skin symptoms; 8- skin tests; 9- exposures; and 10- general health and family size) and was developed for research on occupational skin disease in general or on hand and forearm eczema in particular. It is a set of questions that can be used according to the research needs and applied to a specific population.^1^

It is important to emphasize that all the questions of the NOSQ-2002/S are contained in the NOSQ-2002/L and therefore the translation of the long version of this questionnaire also generates its short version.

The study was approved by the Institution’s Ethics Committee for Research involving Human Participants, CAAE: 31773020.9.0000.5440, opinion nº. 4.303.248, and participants signed an informed consent form.

### TRANSLATION PROCESS AND CROSSCULTURAL ADAPTATION

This project was initiated with the creation of the study review group. The members were chosen based on their knowledge and experience on dermatological disorders and on their experience in producing cross-cultural adaptations of patient-reported outcome measures.

The study review group, comprising four members, was responsible for supervising and coordinating the adaptation process, as well as for producing the different consensus versions.

The process of translation and cross-cultural adaptation of the NOSQ-2002 from English to Brazilian Portuguese was conducted following recommendations from document of the International Society for Pharmacoeconomics and Outcomes Research (ISPOR) named “Principles of Good Practice for the Translation and Cultural Adaptation Process for Patient-Report Outcomes (PRO) Measures.” The translation and cross-cultural adaptation process following ISPOR’s recommendations is composed of 11 steps covered within four phases, as follows.^8^

- **Phase 1** - Two direct translations, first meeting of the study review group, and first reconciled version - Two direct translations into Brazilian Portuguese by native Brazilian translators fluent in English were conducted with the aim of producing a conceptual (but not literal) version that was equivalent to the original. This step was followed by the first meeting of the study review group to ensure that the translation was done in a colloquial and comprehensible language, culminating in the first reconciled version, after harmonization of both translations.
- **Phase 2** - First cognitive interview, second meeting of the study review group, and second consensus version- For the present study, the first cognitive interviews were conducted with volunteers subjects with dermatological diseases, who were invited from the outpatient clinic of atopic dermatitis and chronic urticaria of the University hospital to which the researchers are affiliated (Hospital das Clínicas, Faculdade de Medicina de Ribeirão Preto, Universidade de São Paulo) and a healthy group, composed of employees from different areas of the same hospital. It is noteworthy that, given the circumstances of the COVID-19 pandemic, the interviews took place by video call. Cognitive interviews were conducted to determine volunteers’ understanding of technical words like eczema and urticaria, and to determine the difficulty in answering the questionnaire. The aim of this step was to foster adaptations in the translation to make the questionnaire more comprehensible.^1^ A trained researcher conducted the interviews and, after a detailed explanation of the characteristics of the questionnaire and the purpose of the study, informed consent was obtained from patients and healthy individuals. The interviews were conducted by the same researcher (an undergraduate medical student who received guidance and training to apply the questionnaire) in a structured way through telephone calls. After completing each section of the questionnaire, the volunteers were asked what they had understood regarding the instructions, questions, and answer options, as well as the reason why they had chosen a particular answer.^9^ During the interviews, volunteers’ comments on the questionnaire were recorded; furthermore, popular expressions and any difficulties in understanding were identified. Each interview lasted between 20 and 30 minutes, and volunteers’ comments were later evaluated by the study review group. A second meeting of the study review group took place, and all comments done by the volunteers were discussed and evaluated to create a second consensus version.
- **Phase 3** - Back-translation into English and review of the back-translation by the questionnaire developers - A back-translation of the second consensus version into English was carried out and forwarded to the developers of the original version, with their consent to continue with the translation process. After this step, the study review group decided to conduct a second cognitive interview, with the same characteristics, except for the group of volunteers, which was composed by 10 different volunteers with dermatological diseases.
- **Phase 4** - Third meeting of the study review group and final consensus version - The study review group had a third meeting to analyze volunteer’s answers and comments after the second cognitive interview. After the study review group discussion, the demand for new modifications was not identified, and the final consensus version was established.

## RESULTS

Twenty-eight volunteers were included in the first round of interviews, and their characteristics are described in Table 1. Based on previous studies, the total number of participants was considered higher than that recommended to reach the purpose of the cognitive interview.^1,9^

**Table 1:** Characteristics of volunteers who took part in the first cognitive interview

Patient ID	Skin disease	Age (years)	Gender	Education level
1	Eczema	19	Female	Primary school
2	Eczema	21	Female	High school
3	Eczema	28	Male	Middle school
4	Eczema	41	Female	Middle school
5	Atopic dermatitis	45	Male	High school
6	Eczema	61	Male	Middle school
7	Eczema	65	Female	Middle school
8	Eczema	22	Female	High school
9	Eczema	30	Female	Higher education
10	Eczema	53	Male	Higher education
11	Atopic dermatitis	63	Male	High school
12	Atopic dermatitis	59	Female	Higher education
13	Urticaria	24	Male	High school
14	Urticaria	42	Male	High school
15	Urticaria	20	Female	High school
16	Urticaria	62	Male	Middle school
17	None	71	Female	High school
18	None	77	Male	High school
19	None	57	Female	Higher education
20	None	65	Female	Vocational education
21	None	30	Female	Vocational education
22	None	47	Female	High school
23	None	56	Male	Higher education
24	None	22	Male	Higher education
25	None	59	Female	Higher education
26	None	20	Male	High school
27	None	19	Male	High school
28	None	50	Male	Higher education

### FIRST MEETING OUTCOME

Fourteen out of the 57 questions that comprised the NOSQ-2002/L (24.6%) were classified as equivalent questions (i.e., their literal translations were deemed as sufficient and did not undergo modifications in the process of translation and cross-cultural adaptation-).

Forty-three questions (75.4%) underwent changes at the first meeting of the study review group. The new expressions and words resulting from this process are in bold quoted text in Table 2. For example: we substituted the word “pulse(s)” by “wrist(s),” since the former means “heartbeat” and the latter has our aimed anatomical meaning, in Brazilian Portuguese.

**Table 2:** Modifications made at the first meeting of the study review group (first reconciled version - Phase 1)

Item in English		Item in Brazilian Portuguese: modified consensus version	
Inclusion of the complementary question G4X		G4X. Você é estudante de: graduação, mestrado, doutorado ou pós-doutorado	
G7	How many hours per week do you work in your main job (on average)?	G7.	Quantas horas por semana você trabalha no seu serviço principal (em média)?
G8.	Do you perform any other paid work regularly?	G8.	Você faz algum outro trabalho remunerado regularmente?
A1.	Have you ever had an itchy rash that has been coming and going for at least 6 months, and at some time has affected skin creases? (by skin creases we mean folds of elbows, behind the knees, fronts of ankles, under buttocks, around the neck, ears, or eyes)	A1.	Você já teve vermelhidão como irritação da pele e coceira (eczema) que aparece e desaparece por pelo menos 6 meses, e que em algum momento afetou dobras da pele? (por dobras da pele, entendemos dobras dos cotovelos, atrás dos joelhos, frente dos tornozelos, embaixo das nádegas, ao redor do pescoço, orelhas ou olhos)
A2.	Have you ever had "hay fever’’ or other symptoms of nasal allergy, e.g. from pollens or animals?	A2.	Você já teve rinite alérgica ou outros sintomas nasais de alergia, por exemplo de pólens ou animais?
D1.	Have you ever had hand eczema?	D1.	Você já teve vermelhidão como irritação da pele e coceira (eczema) nas mãos?
D2.	Have you ever had eczema on your wrists or forearms (excluding fronts of elbows)?	D2.	Você já teve eczema em punhos ou antebraços (excluindo as dobras dos cotovelos)?
D3.	Shade areas on the hands or forearms where you commonly have eczema: inclusion of the names of the areas of the hands and forearms in the figure.	D3.	Pinte as áreas das mãos ou antebraços em que você geralmente tem eczema: inclusão dos nomes de áreas das mãos e antebraços, na figura.
	Changes:		Mudanças:
	finger webs		juntas dos dedos
	palms		palmas das mãos
	wrists		punhos
D4.	How often have you had eczema on your hands, wrists, or forearms?	D4.	Com que frequência você tem eczema nas mãos, punhos ou antebraços? (uma resposta em cada coluna, se apropriado)
	only once and for less than two weeks		só uma vez com duração menor que duas semanas
D5.	When did you last have eczema on your hands, wrists, or forearms? (one answer in each column if applicable)	D5.	Quando foi a última vez que você teve eczema em mãos, punhos ou antebraços? (uma resposta em cada coluna, se apropriado)
D6.	When did you last have eczema on your hands, wrists, or forearms? (one answer in each column if applicable)	D6.	Quando você teve eczema nas mãos, punhos ou antebraços? (uma resposta em cada coluna, se apropriado)
D7.	What do you think was the cause of the eczema on your hands, wrists, or forearms when it started?	D7.	0 que você acha que causou (foi a causa) o eczema em suas mãos, punhos ou antebraços quando isto começou?
D10. Have you visited a doctor as an adult for your hand or wrist/forearm eczema?		D10. **Você consultou** um médico quando adulto para o seu eczema em mão ou punho/antebraço?	
D11.	During which season do you have most problems with hand or wrist/ forearm eczema? (one or more answers in each column if applicable)	D11.	Durante qual estação você tem mais problemas com eczema em mãos ou punhos/antebraços? (uma ou mais respostas em cada coluna se apropriado)
D12.	How do you grade your eczema on a scale from 0-10?	D12.	Dê uma nota para seu eczema em uma escala de 0 a 10:
	At worst		no pior momento
F1.	Have you noticed that contact with certain materials, chemicals, or anything else in your work makes your eczema worse? (one answer in each column if applicable)	F1.	Você notou que o contato com certos produtos químicos, materiais ou alguma outra coisa no seu trabalho faz piorar seu eczema? (uma resposta em cada coluna se apropriado)
F2.	Have you noticed that contact with certain materials, chemicals, or anything else outside your work makes your eczema worse? (one answer in each column if applicable)	F2.	Você notou que o contato com certos produtos químicos, materiais ou alguma outra coisa fora do seu trabalho faz piorar seu eczema? (uma resposta em cada coluna se apropriado)
F3.	What do you consider as the most important things outside the workplace that worsen your eczema? (mark no more than 5 things in each column)	F3.	Dentre os fatores abaixo, quais você considera mais importante para a piora do seu eczema fora do trabalho? (não marque mais de 5 itens em cada coluna)
	detergents and other household cleaning and laundry products		sabão em pó e outros produtos de limpeza de casa e lavanderia;
	frequent hand washing		lavagem das mãos muitas vezes
	machine maintenance (e.g. cars), handling oils		cuidar de máquinas (por exemplo, carros), manipular óleos
	construction work, painting, wall-papering, renovation, and decoration		trabalho de construção, reforma, pintura, papel de parede e acabamento
	gardening, handling plants, soil, vegetables, berries, fruits, etc.		jardinagem, horta, terra, pomar, plantas, frutos vermelhos, frutas etc.
	mood, stress		problemas emocionais, estresse
F4.	Does your eczema improve when you are away from your normal work (for example weekends or longer periods)?	F4.	Seu eczema/alergia melhora quando você se afasta do seu trabalho (por exemplo finais de semana ou períodos mais longos)? (uma resposta em cada coluna se apropriado)
C1.	Has eczema on your hands, wrists or forearms affected your daily activities in your occupation in any way? Which of the following statements are true? (mark any that are applicable) not at all ... I have had difficulties in getting a job ... I have been sick-listed or otherwise off work For how long during the past 12 months have you been sick-listed or otherwise off work due to eczema? ^*^ This statement can be left out in work site and occupational surveys.	C1.	0 eczema em suas mãos, punhos ou antebraços atrapalha de alguma maneira suas atividades em sua ocupação? Quais das seguintes afirmações são verdadeiras? (Marque todas que sejam apropriadas) não **atrapalha** de jeito nenhum ... eu tenho dificuldades para conseguir um emprego **... eu fui afastado por doença ou dispensado do trabalho** **Por quanto tempo nos últimos 12 meses você foi afastado por doença ou dispensado** do trabalho devido ao eczema? ^*^ Esta declaração pode ser omitida no local de trabalho e em **pesquisas ocupacionais.**
C2.	How has your eczema affected your life during the past 12 months? (one answer in each line) No effect Slight effect Moderate effect Large effect Not relevant housework, daily activities other hobbies or activities getting about, travel social activities close personal relations sex life mood	C2.	Como o seu eczema afetou sua vida **nos** últimos 12 meses? (uma resposta em cada linha) Não **afetou** **Afetou** pouco **Afetou moderadamente** **Afetou muito** **Não é importante** tarefas domésticas, **atividades de vida diária** **no lazer** ou outras atividades **nos passeios** e viagem **nas** atividades sociais **nas** relações pessoais **íntimas** na vida sexual no humor
C3.	Has your eczema had a negative influence on your financial situation (medical and other linked expenses, lost workdays, work capacity and/ or change of job)? (only one answer) no negative financial effects (no expenses or I have full compensation)	C3.	Seu eczema teve influência negativa na sua situação financeira **(gastos médicos ou com saúde,** dias de trabalho perdidos, mudança de emprego ou sua capacidade para trabalhar)? (Somente uma resposta) sem efeitos negativos (sem gastos ou **tenho reembolso de tudo)**
U1.	Have you ever had itchy wheals appearing and disappearing rapidly (within hours) on your hands, wrists, or forearms (urticaria or nettle rash)?	U1.	Você já teve **inchaço** com coceira **da pele que aparece e desaparece** rapidamente (dentro de horas) em mãos, punhos ou antebraços (urticária ou **reação a urtiga)?**
U2.	Have these itchy wheals (urticaria) on your hands, wrists or forearms been caused by skin contact with fruits, vegetables, rubber gloves, animals, etc.? (wheals appearing in minutes after contact)	U2.	Esse **inchaço** com coceira (urticaria) nas mãos, punhos ou antebraços **foi** causado pelo contato da pele com frutas, vegetais, luvas de borracha, animais etc.? **(urticária** aparecendo em minutos após o contato)
U3.	How often have you had these itchy wheals (urticaria) on your hands, wrists, or forearms? (only one answer)	U3.	Com que frequência você **tem** este **inchaço** com coceira (urticária) nas mãos, **punhos** ou antebraços? (Somente uma resposta)
U4.	When did you last have these itchy wheals (urticaria) on your hands, wrists, or forearms? (only one answer) during the past 7 days	U4.	Quando **foi** o último **inchaço** com coceira (urticária) em suas mãos, **punhos** ou antebraços? (Somente uma resposta) nos últimos 7 dias
U5.	When did you first get these itchy wheals (urticaria) on your hands, wrists, or forearms? (only one answer) below 6 years of age above 18 years of age	U5.	Quando você **teve pela primeira vez** esse **inchaço** com coceira (urticária) em suas mãos, **punhos** ou antebraços? (Somente uma reposta) **menos** de 6 anos de idade **mais** de 18 anos de idade
U6.	What was your occupation when you started having the itchy wheals (urticaria)?	U6.	Qual era sua ocupação quando você começou a ter **inchaço** com coceira (urticária)?
U7.	What was your major activity at work when you started having the itchy wheals (urticaria)?	U7	Qual era sua atividade principal no trabalho quando você começou a ter **inchaço** com coceira (urticária)?
U8.	Have you visited a doctor as an adult because of the itchy wheals (urticaria)?	U8.	**Você consultou médico na idade adulta** por causa do inchaço com coceira (urticária)?
U9.	How do you grade the itchy wheals (urticaria) on a scale from 0-10? (put a mark on the line corresponding to the severity of the urticaria) At worst	U9.	**Dê uma nota você para o seu inchaço** com coceira (urticária) em uma escala de 0 a 10: (coloque a marca na linha que corresponde à gravidade da urticária) **no pior momento**
S1.	Have you had any of the following symptoms on your hands or wrist/ forearms during the past 12 months? (mark in each column any that are applicable) no symptoms during the past 12 months weeping or crusts tiny water blisters (vesicles) papules rapidly appearing itchy wheals/welts (urticaria) burning, prickling, or stinging something else, what?	S1.	Você teve algum dos seguintes sintomas em suas mãos ou punhos/antebraços durante os últimos 12 meses? (Marque em cada coluna qualquer que seja apropriado) sem sintomas nos últimos 12 meses secretando ou crostas bolhas minúsculas com água (vesículas) inchaço localizado da pele (pápulas) aparecimento rápido de inchaço com coceira/vergões (urticária) queimando, formigando ou picando algum outro, qual?
S2.	Do you get a rash from metal buttons, metal fasteners, metal costume jewelry (for example earrings) or other metal objects next to your skin? (apart from under rings)	S2.	Você tem irritação da pele por causa de botões metálicos, fechos metálicos, bijuterias metálicas (por exemplo, brincos) ou outros objetos metálicos encostados na pele? (além dos anéis)
S5.	Have you ever had eczema "on the fronts of the elbows or behind the knees”?	S5.	Você já teve vermelhidão como irritação da pele e coceira (eczema) nas dobras dos cotovelos ou atrás dos joelhos?
T1.	Has a doctor ever diagnosed you with an allergy?	T1.	Você já teve diagnóstico de alergia feito por médico?
T3.	Was the allergy/were the allergies diagnosed with ... patch-tests (tests are normally taped onto the upper back and removed after 1-2 days) skin-prick-tests (test drops are normally placed on the forearm and pricked through with lancets or needles. The results are read after 15-30 minutes)	T3.	A(s) alergia(s) foi (foram) diagnosticadas com... teste de contato (normalmente cola-se a fita na parte superior das costas e retira-se após 1-2 dias) testes de picada na pele (normalmente o teste coloca gotas no antebraço e punciona (fura) com lancetas ou agulhas. Os resultados são lidos após 15 a 30 minutos)
E2.	What type of gloves do you (or did you) use in your work? (mark any that are applicable in each column) cotton gloves underneath rubber or plastic gloves	E2.	Qual tipo de luvas você usa (ou usava) no seu trabalho? (marque qualquer que seja apropriada em cada coluna) luvas de algodão sob luvas plásticas ou de borracha
E3.	Have you had skin symptoms as a result of wearing protective gloves? any gloves	E3.	Você já teve sintomas de pele por ter usado luvas de trabalho? qualquer luva
E5.	What are you doing or handling in your work at present? (one or more answers) preparing food/handling food cleaning agents paints, lacquers, coatings, etc. sealants, putty, plaster, flooring agents, cement etc. dust (wood dust, grinding dust, paper dust etc.) soil, waste, or other dirt	E5.	0 que você faz ou manipula em seu trabalho atualmente? (uma ou mais respostas) preparar/manipular alimentos produtos de limpeza tintas, vernizes, laquês etc. selantes, massa de vidraceiro, gesso, cola de piso, cimento etc. poeira (de madeira, de moagem, de papel etc.) terra, lixos ou outra sujeira
E6.	How many hours per day do you currently do the following activities outside of your work? (mark any that are applicable, make your best estimate) cleaning or washing	E6.	Quantas horas por dia você atualmente faz as seguintes atividades fora do seu trabalho? (marque qualquer que seja apropriada, faça sua melhor estimativa) limpar ou lavar
E7.	How often did you do the following activities outside of your work during the past 12 months? (mark any that are applicable, make your best estimate) gardening (in the season) car or motor repair hobbies	E7.	Com que frequência você realizou as seguintes atividades fora do seu trabalho nos últimos 12 meses? (marque qualquer que seja apropriada, faça sua melhor estimativa) jardinagem conserto de automóveis ou motores lazer
E8.	How many times do you wash your hands during a usual working day? (include hand washing during your work and at home/outside work)	E8.	Quantas vezes você lava as mãos durante um dia comum de trabalho? (Inclua lavar as mãos durante o trabalho e fora do trabalho/em casa)
H1.	Would you say your overall health, as compared to others of your own age, is... (only one answer) fair poor	H1.	Você diria que sua saúde geral quando comparado a outros da sua idade, é... (somente uma resposta) razoável ruim

Changes made at the first meeting of the study review group highlighted in bold.

### SECOND MEETING OUTCOME

At the second meeting of the study review group, in which the second consensus version was created, modifications were made to three questions. In question G4, changes were made to the instructions (informative explanation) for those interviewed in the “student” category, which used to be “skip to question G8, page 2” and now read as follows: “if only student, skip to question G8, page 2,” and in the “retired and pensioner” category, which was previously “skip to question A1, page 3” and now reads as follows: “if only retired/pensioner, skip to question A1, page 3”. These modifications were carried out because in Brazil some students and retirees/ pensioners may be simultaneously performing other work activities. Just below question E4 we have included an instruction (informative explanation): “If you are not currently working or are just retired/pensioners, skip to question E6, page 19,” as some respondents were currently unemployed or are retired/pensioners and did not perform any type of work with the activities addressed in question E5.

In question E5, definitions were included for two response categories within the question. The “wet work” category was defined as: “wet work (works with exposure to water, for example, cleaning sectors, restaurant kitchens)” and the “cutting fluids” category was defined as: “cutting fluids (liquid or gas applied to a tool or material to facilitate the cutting operation), etc.” We decided to include definitions and examples for these two categories, as most volunteers did not understand the meaning of these two terms.

Table 3 shows five categories of modifications due to cultural adaptation in this group of questions. Among the 43 modified questions, 10 required two changes (G4, A1, D3, F3, F4, C1, C2, C3, U5 and S1). Most changes were performed to improve understanding in Brazilian Portuguese and not due to difficulties in translation.

**Table 3:** Categories of modifications in the Nordic Occupational Skin Symptoms Questionnaire (NOSQ-2002/L [long version] and NOSQ-2002/S [short version]) during the process of translation and cross-cultural adaptation (first and second version)

Modifications categories	Questions
1- Modified or new instructions (informative explanation)	G4, U5 and below E4
2- Inclusion of a complementary question	G4X
3- Inclusion of definitions and additional information	D1, D3,S1 and E5
4- Inclusion or change of important terms, alternative words or synonyms	A1, F3, F4, C1, C2 and C3
5- Change of words	G7, G8, A1, A2, D2, D3, D4, D5, D6, D7, D10, D11, D12, F1, F2, F3, F4, C1, C2, C3, U1, U2, U3, U4, U5, U6, U7, U8, U9, S1, S2, S5, T1, T3, E2, E3, E5, E6, E7, E8 and H1

After the back-translation, no major modification was necessary, but since minor modifications were included in the final consensus version, the second round of interviews was performed only with 10 different volunteers with dermatological diseases (Table 4), and no relevant suggestion was raised by the interviewees. Moreover, they did not have difficulty in answering the questionnaire, which was considered easy to understand.

**Table 4:** Demographic and clinical characteristics of volunteers who took part in the second round of the questionnaire interview

Patient ID	Skin disease	Age (years)	Gender	Education level
1	Eczema/urticaria	44	Female	Higher education
2	Eczema	28	Male	High school
3	Eczema	39	Female	High school
4	Eczema/urticaria	55	Male	High school
5	Eczema	29	Male	High school
6	Atopic dermatitis	22	Female	High school
7	Urticaria	49	Male	High school
8	Eczema	38	Female	High school
9	Atopic dermatitis	21	Male	High school
10	Eczema	49	Male	Primary school

In the first round of interviews, we found that some interviewees were not aware of the diagnostic methods of skin allergy: patch test, skin prick test, radioallergosorbent tests (RAST), but from the moment the interviewer read the explanations aloud, explaining how each method is performed, the subjects understood the meaning and responded.

## DISCUSSION

We created a Brazilian Portuguese version of the NOSQ-2002/L and NOSQ-2002/S questionnaires through a process of translation and cross-cultural adaptation that was rigorously developed, making it equivalent to the original English version. The method we used was adopted by several countries in Europe to adapt instruments that are essential g, as well as in applicability in clinical practice or in public health surveillance.^1,8,10-12^

Adapting an instrument developed in a different language and culture saves time as opposed to developing a new questionnaire. However, if the adaptation is carried out through a simple translation, it is unlikely that there will be a compatible tool, due to the influence of language and culture on healthrelated issues.^13^ In this context, we believe that it was possible to reach an adequate version of the NOSQ2002/L and NOSQ-2002/S questionnaires, which will allow international comparative studies with other versions, as well as multicenter studies. Furthermore, these instruments should ideally be tested under the psychometric perspective in the future.^14^

The adaptation process of the NOSQ-2002/L questionnaire was relatively straightforward, since the original instrument was made available in English and in technical (professional) language, enabling translation into any language of destination. The simplicity with which most questions were done, as well as the fact that the questionnaire did not use colloquial terms in the original English version, facilitated the cultural adaptation of the instrument.

We had no major difficulties in adapting the instrument to Brazilian Portuguese; the only difficulty that arose during the process was to adapt some items according to the sociocultural behavior of the Brazilian population. It is important to emphasize that some changes implemented to improve understanding of the questionnaire may interfere with the answers. Therefore, it will be extremely important that the results obtained with the English and Brazilian Portuguese versions are compared to test cultural invariance.

Most changes took place at the first meeting of the study review group, culminating in the first reconciled version. After the first cognitive round of interviews, only three modifications were made, culminating in the second consensus version. Although there was no need to conduct the second round of cognitive interviews with volunteers, since the guidelines recommend only one round of cognitive interviews,^8^ the study review group decided to carry it out with the purpose of testing the understanding of the second consensus version. Other strengths of our study were the number of volunteers included and the inclusion of volunteers with and without skin diseases. Thus, we believe that our version can be completed by any of these respondents in research or work environments.

Overall, the interviews confirmed that the level of understanding of the questionnaire was good for all volunteers. However, some modifications were suggested to improve understanding of the language, as well as the inclusion of more colloquial expressions, which allowed better understanding of the questions.

Dermatitis is a group of skin diseases that generates high demand for medical care in Brazil, due to high prevalence; ODs are poorly identified or valued by physicians, who can underestimate their morbidity and dimension as a public health problem. The main causes of ODs are chemical agents, which cause primary irritant contact dermatitis. It is estimated that these agents cause approximately 80% of ODs, which may manifest as an acute, subacute, or chronic inflammatory reaction and affect mainly the upper limbs, especially the hands.^15^

A study on OD conducted in Brazil involved a cohort of 4,710 patients and identified that men aged between 35 and 49 years are the most affected. In addition, the most affected body region was the upper limb (34.2%) and the hand (25.4%).^16^

In order to have efficient prevention measures for occupational allergic diseases, it is essential that early screening is carried out to allow adequate interventions and diagnosis in the initial stages, enabling the treatment to be more efficient.^7^ In Brazil, there are few self-report instruments for occupational skin diseases that specifically address this health condition. For this reason, our study group decided to conduct the process of translation and cross-cultural adaptation of the NOSQ-2002.

Preventive measures, such as guidelines on and replacement of chemical products, adequacy of the work environment, conscientious use of personal protective equipment, are conditions that can be easily evaluated. It is estimated that the number of medical evaluations for detection of skin diseases on hands and contact urticaria could be reduced with the implementation of the NOSQ-2002 by prevention services (internal and external).^1^

The NOSQ-2002 questionnaire is considered a screening and research tool for dermatologists, epidemiologists, public health specialists, and occupational medicine specialists, in addition to being an auxiliary tool for family physicians.^1^ However, it is essential that a psychometric study is carried out before its implementation in clinical, epidemiological, public health, research environments and in occupational medicine.

Therefore, the present study is just the initial step of the process of producing a valid Brazilian Portuguese version of the questionnaire; the next step will be performing rigorous evaluation of the reliability and validity of this version (internal validation), as well as sensitivity and specificity analysis.^17-20^ Lack of internal validation is a limitation of the study.

At the end of the adaptation process, the short and long versions of the NOSQ-2002 questionnaire for the Brazilian Portuguese language are available.
